# Tailoring substrate adhesion *via* flexible chain architecture design in benzoheterocycle polyimide protective coatings

**DOI:** 10.1039/d5ra05693d

**Published:** 2025-10-20

**Authors:** Feng Guo, Wei Wang, Guangtao Qian, Dandan Li, Yongfeng Li

**Affiliations:** a State Key Laboratory of Advanced Fiber Materials, Center for Advanced Low-dimension Materials, College of Materials Science and Engineering, Donghua University Shanghai 201620 China; b School of Materials Science and Engineering, NingboTech University Ningbo 315100 China; c Shanghai Collaborative Innovation Center of High Performance Fibers and Composites (Province-Ministry Joint), Center for Civil Aviation Composites, Donghua University Shanghai 201620 China yfli@dhu.edu.cn; d School of Materials Science and Engineering, Shanghai University of Engineering Science Shanghai 201620 PR China

## Abstract

Benzoheterocycle polyimides (PIs) demonstrate exceptional metal substrate adhesion, positioning them as high-performance alternatives to conventional heterogeneous adhesives. These materials significantly enhance the weather resistance of flexible batteries and facilitate the development of thinner and lighter PI-based aluminum-plastic flexible packaging. Through molecular engineering of benzoheterocycle PI backbones, a series of ternary copolyimides (BIBOPIs) were synthesized utilizing four structurally distinct flexible diamine monomers. The thermodynamic properties, solvent resistance, water absorption, and coating adhesion of BIBOPIs were systematically evaluated. The distinctive molecular architectures of flexible diamines impart unique physicochemical properties to BIBOPIs. The light transmittance of BIBOPIs incorporating 4,4′-diaminodiphenylsulfone (DDS) exceeds 73%, with BIBOPI-0.5DDS achieving a transmittance of 85% at a wavelength of 800 nm. The light transmittance of BIBOPI-0.5RODA is reduced compared to that of BIBOPI-0.3RODA as the content of flexible ether groups in the molecular chain increases, consequently limiting its visible light transmittance. With the incorporation of a third monomer, the glass transition temperature (*T*_g_) values of BIBOPIs demonstrate a consistent decrease, ranging from 332 to 410 °C. With the increase in 1,4-bis(4-aminophenoxy)benzene (RODA) ratio, the elongation at break of BIBOPIs exhibits a significant rise, from 6.8% for BIBOPI-0.1RODA to 33.8% for BIBOPI-0.5RODA. BIBOPIs demonstrate exceptional solvent resistance at both ambient and elevated temperatures. Additionally, the water absorption (*W*_A_) of BIBOPIs decreases as the proportion of the third component increases. The adhesion grade of BIBOPI coatings is 0, with the exception of BIBOPI-0.1DDS, BIBOPI-0.1BPDA, and BIBOPI-0.1ODA. Pull-off experiments demonstrate that the incorporation of a flexible third monomer enhances the adhesion between BIBOPI coatings and substrates, with the adhesion strength surpassing 21.7 MPa. Unlike previous studies, the PI coating developed in this research enables the formation of a metal substrate protective layer with excellent adhesion, achieved through molecular design and structural optimization, without requiring complex surface treatment techniques. The optimized coating architecture enables molecular-level integration with dense PI protective layers, providing critical insights for developing advanced benzoheterocycle PI-based aluminum-plastic flexible packaging systems.

## Abstract 2

Benzoheterocycle polyimides (PIs) demonstrate exceptional metal substrate adhesion, positioning them as high-performance alternatives to conventional heterogeneous adhesives.

## Graphical abstract

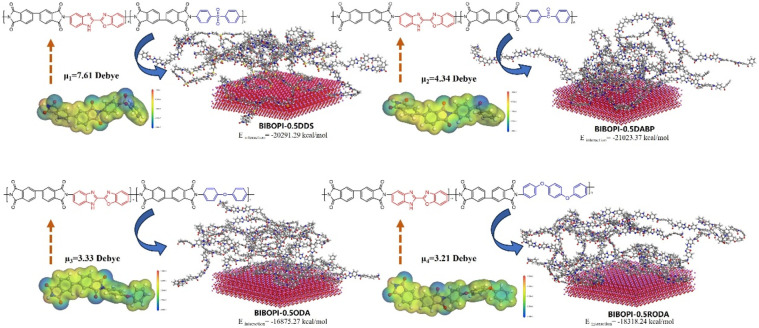

## 1 Introduction

Polyimide (PI) materials represent a class of high-performance polymers characterized by their exceptional mechanical properties, superior chemical resistance, and exceptional thermal stability at elevated temperatures. Additionally, these materials exhibit low coefficients of thermal expansion (CTE) and dielectric constants, rendering them particularly suitable for applications across electronics, information technology, energy systems, and power engineering.^1–5^ Nevertheless, a critical limitation of conventional PI lies in its intrinsically stable surface energy, which compromises interfacial adhesion with metallic substrates. This deficiency poses significant challenges for flexible electronic devices, which are routinely subjected to complex deformation modes such as bending, twisting, and thermal expansion. Particularly concerning is the interfacial stress generated during mechanical bending, which may induce delamination at PI-metal interfaces. Consequently, enhancing the interfacial adhesion between PI and metallic components emerges as a critical requirement for ensuring the long-term operational reliability and durability of flexible electronic systems.^6–10^

In soft-pack battery applications, adhesive resins have been developed to enhance interfacial layer bonding. However, the distinctive three-dimensional network internal structure of cured resins presents inherent material limitations, including brittleness, high cross-linking density, elevated internal stress, diminished toughness, and reduced impact resistance.^11^ These adhesives also demonstrate insufficient environmental stability and face interfacial compatibility challenges due to PI dense structure and chemically inert surface. The bonding interface becomes susceptible to void formation, air entrapment, volatile byproduct generation, and uneven dispersion of reinforcement materials. These defects create microstructural pathways for crack nucleation and propagation, critically undermining interfacial integrity. To address these limitations, surface modification strategies for PI have gained prominence. Physical deposition techniques, such as magnetron sputtering or vapor deposition, enable the application of Cr, Al, Ti, W, or their alloy coatings onto PI films as adhesive interlayers, achieving 2–3 times adhesion enhancement.^12^ The primary limitation is the material's restricted capability to withstand subsequent processing, which may result in the delamination of the metal film deposited on the PI during deformation.

The second approach involves chemical modification of PI substrates, where surface activation efficacy depends critically on both surface roughness and chemical composition. Key methodologies include hydrolytic treatment, plasma or laser processing, and grafting modification. Hydrolysis alters PI surface uniformity by enhancing polarity, hydrophilicity, and roughness which are factors that improve adhesive wettability and metal adhesion.^13^ These treatments increased surface roughness by approximately 84% compared to untreated films, though unpredictable mechanical degradation from hydrolytic chain scission remains a concern.^14^ Plasma and laser techniques provide alternative activation pathways. Carbides and micropores were generated on the surface of the PI substrate through CO_2_ laser pretreatment.^15^ This process not only increased the surface roughness but also significantly enhanced the mechanical interlocking effect. Nevertheless, the processing area is restricted by limitations of the equipment. Regarding the mechanism, the molecularly linked branches exhibit specificity and can be precisely engineered based on the characteristics of the adhesion target. Lin designed the methyl acrylamide group on the upper branch of the heteranthrene ring in PSPI to form a three-dimensional cross-linked network and a distinctive rigid resin structure *via* ultraviolet irradiation.^16^ Chan-Park successfully copolymerized 4-vinylpyridine directly onto a PI film and subsequently laminated the modified PI film onto copper foil under atmospheric pressure without employing a polymerization initiator.^17^ The adhesion between the PI film and the copper foil can be significantly improved. However, the treatment process was both complex and expensive, requiring argon plasma pretreatment of PI films prior to grafting and laminating.

Therefore, given the practical significance of adhesion mechanisms, it is crucial to develop coating materials that can effectively interact with substrates through precisely engineered molecular structures. An Extensive literature review suggests that the rotational energy barrier within the polymer chains can be reduced by the incorporation of flexible components, including carbonyl, sulfone, and ether bonds. This not only increases chain flexibility and the density of mechanical interlocking entanglements but also effectively improves adhesion between the polymer and metal, ceramic, or glass substrates.^18,19^ Furthermore, the incorporation of polar functional groups such as carbonyl or sulfone within the molecular chain facilitates the formation of chemical bonds between the polymer and both metallic and inorganic non-metallic substrates, thereby enhancing complexation.^20–23^ The exceptional thermal stability and low CTE of benzimidazole and benzoxazole, together with their potential interactions with metals, have been the subject of extensive research. It is anticipated that the benzoheterocycle PI film with enhanced adhesion to the substrate can be developed through strategic molecular design, thereby constructing a protective layer of aluminum-plastic flexible packaging. Notably, the superior adhesion of the designed benzoheterocycle PI film facilitates the formation of a tightly infiltration bonding with the highly dense PI protective layer. It not only reduces the dependence on heterogeneous adhesives but also fundamentally enhances the battery's environmental resistance. Furthermore, this advancement facilitates the development of a thinner and lighter PI-based aluminum-plastic flexible packaging, which possesses substantial practical value. However, research in this field has been limited. None of the relevant studies have provided detailed information regarding the structural formula, preparation process, or performance characteristics of the PI materials employed, thereby failing to offer sufficient guidance for their application in battery outer protective layers.

To reduce the dependence of protective coatings on heterogeneous adhesives and significantly enhance the environmental weather resistance of batteries, this study developed a benzoheterocycle PI film through precise molecular structure design and the establishment of effective substrate interactions, thereby constructing an aluminum-plastic composite soft-pack protective layer. Following an initial experimental investigation and a comprehensive assessment of the physicochemical properties of benzoheterocycle rings, benzimidazole-benzoxazole diamine (BIBO) was selected in this study as the subject for further investigation.^24^ The flexible diamine monomers, including DDS, 4,4′-diaminobenzophenone (DABP), 4,4′-oxydianiline (ODA), and RODA, were integrated into the molecular framework. By adjusting the ratio of BIBO to these monomers, the molecular chain structure of PI was systematically controlled. The influence of the third monomer structure on the adhesion properties of BIBOPIs was more comprehensively illustrated through simulation. The results demonstrated a significant increase in the elongation at break with the elevation of the RODA ratio. BIBOPI films exhibited superior solvent resistance under both ambient and elevated temperature conditions. Additionally, BIBOPI coated aluminum substrates preserved their fundamental morphology in electrolytic environments. With the increase in the specific gravity of the third component, the water absorption of the film correspondingly decreased, which positively influenced the preparation of the composite material. The adhesion performance between the BIBOPI coating and the substrate was thoroughly evaluated by utilizing a combination of grid tests, pull-off tests, and boiling tests. The efficient adhesion of PI coatings could be achieved through simple coating and controlled thermal programming. For the BIBOPI coatings, the adhesion strength can be significantly enhanced through the incorporation of a small amount of RODA. The adhesion strength of BIBOPI-0.5RODA reached 33.3 MPa, which markedly surpassed that of the BIBOPI coatings composed of DDS, DABP, and ODA components. It was noteworthy that the designed benzoheterocycle PI film exhibited excellent adhesion. It not only facilitated the formation of a strong wetting bond with the high-density PI protective layer but also enabled activation of the coating structure through simple interfacial modification techniques, thereby achieving further functionalization. This advancement not only contributes to the expansion of battery application environments, but also supports the development of thinner and lighter aluminum-plastic composite flexible packaging materials, demonstrating considerable commercial potential.

## 2 Experimental section

### 2.1 Materials

4,4′-Biphthalic anhydride (BPDA, 98%), *N*-methyl-2-pyrrolidone (NMP, Water ≤ 50 ppm, 99.8%), 4,4′-diaminodiphenylsulfone (DDS, 99%), 4,4′-diaminobenzophenone (DABP, 98%), 4,4′-oxydianiline (ODA, 98%) and 1,4-bis(4-aminophenoxy)benzene (RODA, 98%) were commercially available and used without further purification. BIBO was prepared by referencing previously published literature.^24^

### 2.2 Preparation of BIBOPI film

**Scheme 1:**
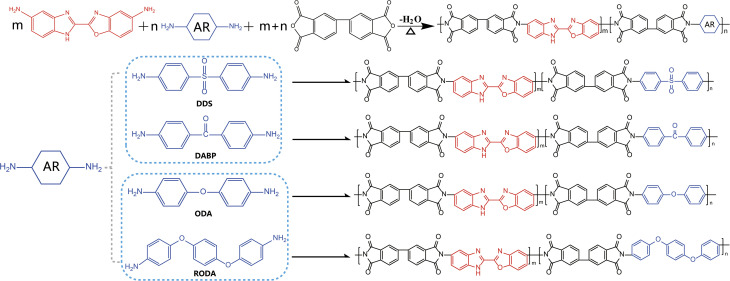
Preparation routes of BIBOPIs.

In this study, the lab-synthesized BIBO required pre-drying prior to use, while commercially obtained monomers (BPDA, DDS, DABP, ODA, and RODA) were employed directly without additional purification. BIBOPIs were synthesized through a conventional two-step thermal imidization process, as schematically illustrated in Scheme 1. Taking BIBOPI-0.5DDS as a representative example, the synthetic procedure was described as follows. A precisely weighed mixture of BIBO (0.2642 g) and DDS (0.2483 g) was dissolved in anhydrous NMP (1.3031 g) under vigorous stirring in an ice-water bath (0–5 °C) until the diamine was completely dissolved. BPDA (0.2942 g) was then gradually introduced to the homogeneous diamine solution. The reaction system was maintained in the ice bath with continuous mechanical stirring for 6 h, followed by an additional 14 h of polymerization at ambient temperature. Throughout the reaction process, periodic additions of anhydrous NMP were made to maintain appropriate solution viscosity and ensure reaction homogeneity. The resultant polyamidic acid (PAA) precursor solution was subsequently subjected to vacuum degassing for 5 h to eliminate entrapped air bubbles prior to further processing.

A uniformly thick PAA solution was cast onto a horizontally positioned clean glass plate using a precision doctor blade set at 300 μm gap thickness, ensuring uniform film formation. The cast film underwent a staged thermal imidization protocol: initial solvent evaporation was achieved through isothermal drying in a convection oven at 80 °C for 12 h. The sample was then transferred to a programmable vacuum oven for sequential thermal imidization under controlled conditions. The temperature profile consisted of four distinct stages: 100 °C (1 h), 200 °C (1 h), 300 °C (1 h), and 400 °C (1 h). Following thermal treatment, the system was allowed to cool gradually to room temperature under vacuum. The fully imidized BIBOPI film was liberated from the substrate through hydrothermal separation in deionized water at 80 °C. The BIBOPIs were systematically designated based on diamine composition: BIBOPI-*x*DDS denotes materials where *x* represents the molar fraction of DDS relative to total diamine content (*x* = 0.1, 0.3, 0.5 corresponding to 10%, 30%, and 50% molar incorporation respectively). Analogous naming conventions were applied to derivatives incorporating DABP, ODA, and RODA as co-monomers, maintaining consistent stoichiometric notation.

### 2.3 Fabrication of BIBOPI-coated aluminum substrate

The BIBOPI-coated aluminum substrates were prepared, and the adhesion and solvent resistance of the BIBOPI coatings were investigated. Aluminum-based materials (H1008) are widely available commercial products, typically fabricated in disc form with an approximate thickness of 60 μm and a radius of around 3 cm. Aluminum substrates underwent surface activation by immersion in a 2 wt% NaOH solution at 70 °C for 1.5 min to remove residual contaminants. After confirming that the surface was completely dry, the PAA was uniformly deposited onto the pretreated aluminum substrate *via* spin-coating at a speed of 1000 r min^−1^ for 60 s. The PAA-coated aluminum substrates were placed in a vacuum oven for 5 h and then transferred to an oven maintained at 80 °C for 8 h. Ultimately, the substrates were moved to a vacuum oven for predetermined thermal treatment. The heating process followed the procedure outlined in Section 2.3. Afterward, the BIBOPI-coated aluminum substrates were allowed to cool naturally to ambient temperature in a controlled manner. The thickness of the obtained PI coating ranged from 15 to 20 μm.

### 2.4 Characterization methods

The FTIR spectrum of BIBOPIs was obtained using a Bruker Tensor II spectrometer with 32 scans. Through attenuated total reflection (ATR) infrared attachment analysis, the test wavenumber range was 4000–400 cm^−1^. The UV-Vis spectrum was recorded using the Shimadzu UV-3600 spectrophotometer over a wavelength range of 200–800 nm under transmission mode, where the film thickness was approximately 25 μm. The contact angle of BIBOPIs was measured utilizing the XG-CAMC3 goniometer (Xuanyi Chuangxi Industrial Equipment, Shanghai, China) at 25 °C, using ultra-pure water was served as the liquid medium. A high-resolution ion sputtering instrument (208HR) was employed to deposit a gold coating on the BIBOPI films for 60 s. The morphological characteristics of the coated film were analyzed at ambient temperature using field emission scanning electron microscopy (SEM, Regulus8230, HITACHI, Japan). The thermal stability and degradation behavior of BIBOPIs were investigated within the temperature range of 50–800 °C using the Discovery TGA 550 instrument (TA Instruments, USA), under N_2_ and air atmosphere, at a heating rate of 10 °C min^−1^ and a sample mass of 5–10 mg. The Differential Scanning Calorimetry (DSC) analyses were conducted using the Discovery 250 differential scanning calorimeter (TA Instruments, USA) across a temperature range of 50–400 °C under a N_2_ atmosphere, with a heating rate of 10 °C min^−1^ and a sample mass of 5–10 mg. The dynamic mechanical analysis (DMA) of the BIBOPIs was performed using the RSA-G2 instrument (TA Instruments). The film was subjected to stretching at a frequency of 1 Hz, an amplitude of 20 μm, and a heating rate of 5 °C min^−1^. Static thermodynamic analysis of the film was conducted utilizing a TMA Q400 (TA Instruments) under a N_2_ atmosphere. The analysis utilized a film/fiber fixture with a preload of 0.1 g μm^−1^ and a heating rate of 10 °C min^−1^. The coefficient of thermal expansion (CTE) values for the samples were determined over the temperature range of 50–250 °C. The mechanical properties of the BIBOPI films were assessed utilizing a universal testing machine (Instron 5966), employing a film stretching fixture. The dimensions of samples were 50 mm in length, 5 mm in width, and approximately 25 μm in thickness. The strain rate was set at 5 mm min^−1^, and each sample was subjected to five replicate tests under ambient temperature.

Prior to evaluating the *W*_A_ of the BIBOPI films, the specimen was subjected to drying in a vacuum oven at 150 °C for 24 h. The BIBOPIs were subsequently immersed in deionized water at a temperature of 25 °C. The weight gain of the sample was measured after 48 h, and the *W*_A_ was calculated based on the weight difference. The calculation formula is as follows:
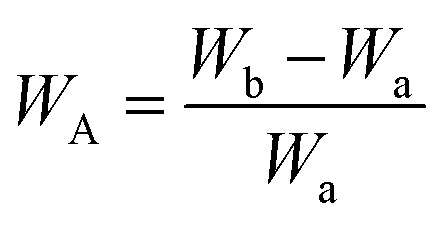
where *W*_a_ represents the quality of the dry BIBOPI film, and *W*_b_ represents the quality of the BIBOPI after surface water has been wiped.

The BIBOPI films were immersed in an organic solvent with a concentration of 10 mg mL^−1^ to examine dissolution behavior. The phase identification of the BIBOPIs was performed using a DX-2700B X-ray diffractometer (XRD) operated at 40 kV and 30 mA, utilizing Cu as the target material. The adhesion of the BIBOPI coating was assessed in accordance with the grid test method specified in ISO 2409. This standard required the use of a guiding device with 1 mm spacing to apply consistent force to the cutting tool, allowing six precise incisions to be made on the coated substrate at a uniform speed. This procedure was repeated to create six additional parallel incisions, forming a grid pattern that intersects at 90° relative to the initial set of incisions. Subsequently, a high-strength adhesive tape was applied to the surface, and the coating was uniformly peeled off in a single direction at a constant speed to evaluate whether the coating detached from the substrate. Five tests were performed on each BIBOPI sample to assess the level of adhesion, ranging from 0 (representing minimal shedding areas and good adhesion) to 5 (indicating extensive shedding areas and poor adhesion). A metal spindle with a diameter of 20 mm was employed for the adhesion strength test. The epoxy composite adhesive was applied to both surfaces of the sample through a scraping method, and then the sample was overlaid onto the opposing metal spindle. The resin was initially cured at 25 °C for 12 h, followed by post-curing in an electrically heated convection oven at 60 °C for 4 h. The adhesion strength was evaluated using a universal testing machine, with three specimens tested per group. The mean values obtained from these tests were recorded as the experimental results. The steady-state molecular structure and net charge were calculated using the GGA-BLYP/DND method *via* the Dmol3 module in Materials Studio 2020. To investigate the interaction between BIBOPI films with varying structures and substrates, molecular models were constructed and simulations were conducted using the Forcite module. The COMPASS II force field was selected for modeling, after which annealing and dynamic optimization were performed under NPT ensemble conditions.^25–27^

## 3 Results and discussion

**Fig. 1:**
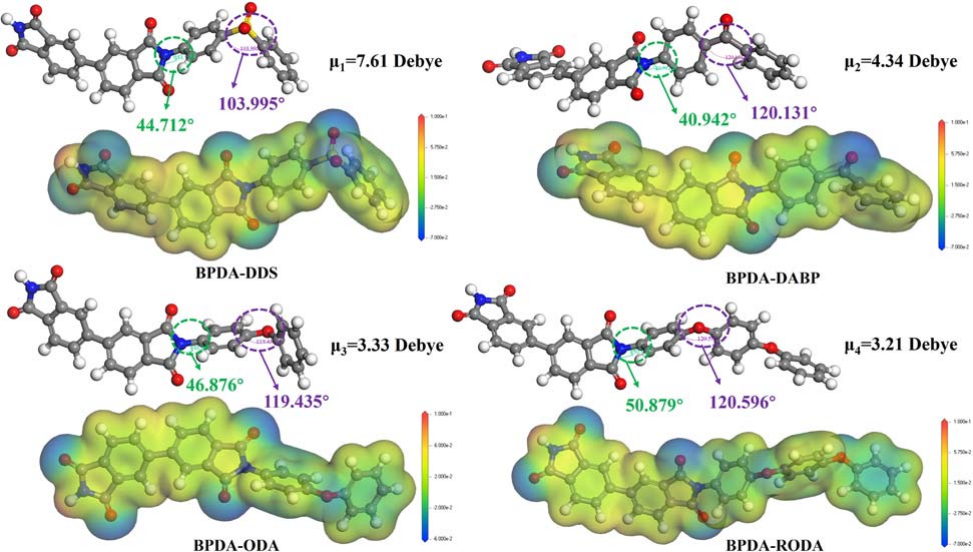
Optimized geometry and electrostatic potential surfaces of BPDA-DDS, BPDA-DABP, BPDA-ODA and BPDA-RODA polymer structures (color code: H atom, white; C atom, dark gray; N atom, blue; S atom, yellow; O atom, red).

In order to gain a more intuitive understanding of the impact of the third monomer on the spatial structure of the molecule,the molecular geometries and electronic properties of BPDA-based PI (DDS, DABP, ODA, RODA) were computationally investigated utilizing the Dmol3 module in Materials Studio 2020 with the GGA-BLYP/DND methodology. As illustrated in Fig. 1, diamine flexibility significantly modulated the dihedral angle between imide rings and adjacent phenyl groups. BPDA-ODA (46.876°) and BPDA-RODA (50.879°) exhibited greater torsional distortion compared to BPDA-DABP (40.942°), while BPDA-DDS demonstrated an intermediate twist (44.712°) with a characteristic sulfone-phenyl bond angle of 103.995°. This folded conformation suggesting increased polymer chain free volume. These structural changes may alter the microstructure of BIBOPIs, consequently impacting their barrier properties. In the simulation results, the bond angles between the carbonyl and ether groups and the benzene ring were very similar. Furthermore, the surface electrostatic potential distributions of these materials were also analyzed. Fig. 1 clearly demonstrated that the electron density distributions of BPDA-ODA and BPDA-RODA were relatively uniform. In contrast, the electron density distribution of BPDA-DDS was non-uniform, primarily attributed to the lone pairs of electrons on the oxygen atoms. BPDA-ODA and BPDA-RODA exhibited comparable dipole moments. The incorporation of sulfone and carbonyl groups significantly enhanced molecular polarization. The dipole moments of BPDA-DDS and BPDA-DABP were 7.61 debye and 4.34 debye, respectively.^28^

**Fig. 2:**
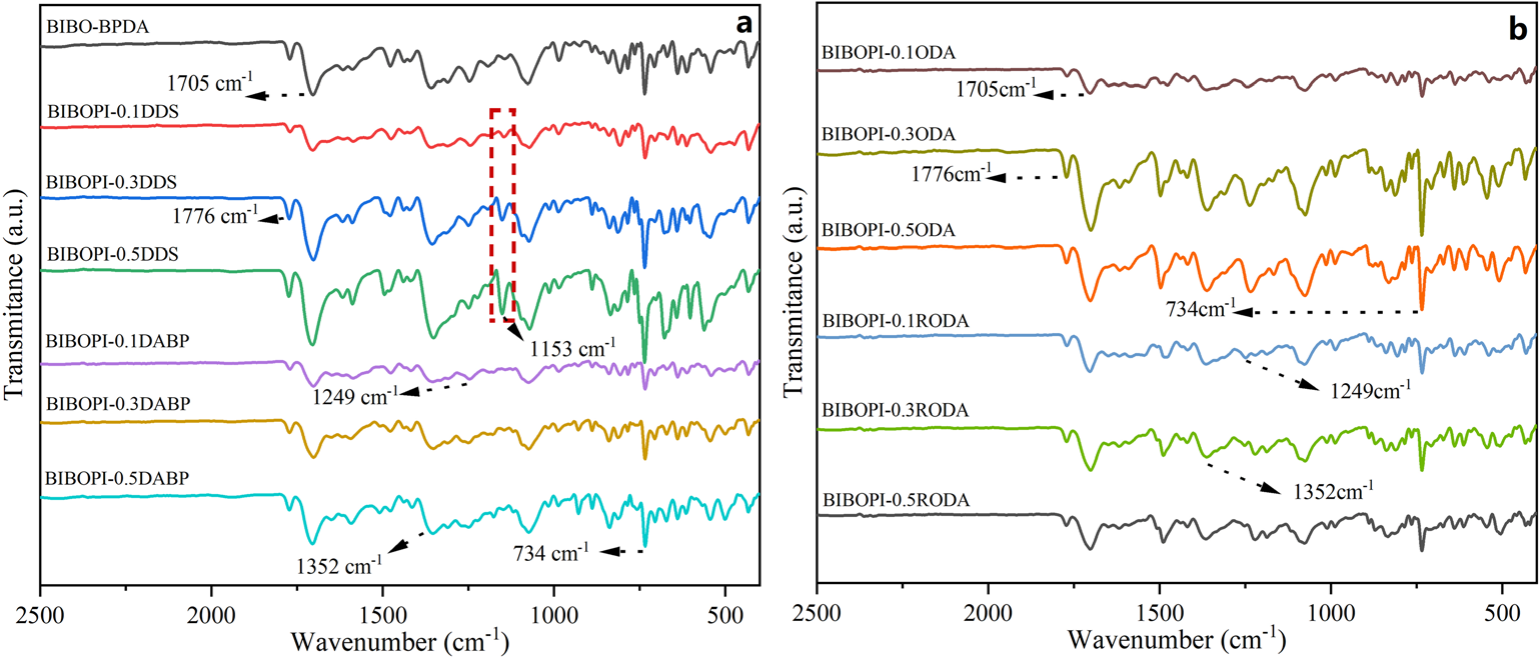
FTIR spectra of (a) BIBOPIs containing DDS and DABP diamine, (b) BIBOPIs containing ODA and RODA diamine.

FTIR analysis provided quantitative verification of successful imidization and structural integration in BIBOPI films, as shown in Fig. 2a and b. The BIBOPI films obtained through thermal imidization exhibited characteristic spectral bands at 1776 cm^−1^ and 1705 cm^−1^, corresponding to the asymmetric and symmetric stretching vibration absorption peaks of the C=O bond, respectively.^29^ The stretching vibration of the C–N bond within the imine ring was observed at 1352 cm^−1^, while the absorption peak at 734 cm^−1^ was attributed to the bending vibration of the imine ring.^30,31^ The absence of residual PAA signatures (1660 cm^−1^, 1550 cm^−1^) confirmed complete cyclization. Notably, the 1249 cm^−1^ band corresponding to Ar–C–O asymmetric stretching validated BO unit incorporation into the PI backbone. Comparative analysis revealed sulfone group integration through emerging O=S=O vibrations at 1153 cm^−1^, with intensity progression (BIBOPI-0.1DDS < 0.3DDS < 0.5DDS) demonstrating concentration-dependent incorporation efficiency of polar sulfonyl moieties, thereby confirming precise control over the designed PI architecture.

UV-Vis spectroscopic analysis revealed modified optical properties in BIBOPI films through strategic monomer incorporation, as shown in Fig. S1a and b. The strong electron-withdrawing sulfonyl groups effectively suppressed charge transfer complex (CTC) formation by reducing chromophore density,^32,33^ yielding enhanced transparency (*T*_800nm_ ≥ 78% for DDS-containing variants) with BIBOPI-0.5DDS demonstrating optimal performances (*T*_800nm_ = 85% and *λ*_cutoff_ = 416 nm), in contrast to the other components which exhibited a minimum *λ*_cutoff_ of 437 nm. The incorporation of ODA can disrupt the regularity of polymer molecular chains and influence the conjugation length of the main chains. Therefore, as the concentration of ODA components increased, the light transmittance of the BIBOPIs also demonstrated a progressively upward trend. The light transmittance of the BIBOPI film was improved as a result of incorporating a minor amount of RODA components. However, as the proportion of flexible ether group components in the molecular chain increased, the long-range molecular chain acquired a degree of flexibility. This enhanced flexibility facilitated inter-chain stacking, thereby limiting the transmission of visible light. The light transmittance of BIBOPI-0.5RODA was considerably lower than that of BIBOPI-0.3RODA.

**Fig. 3:**
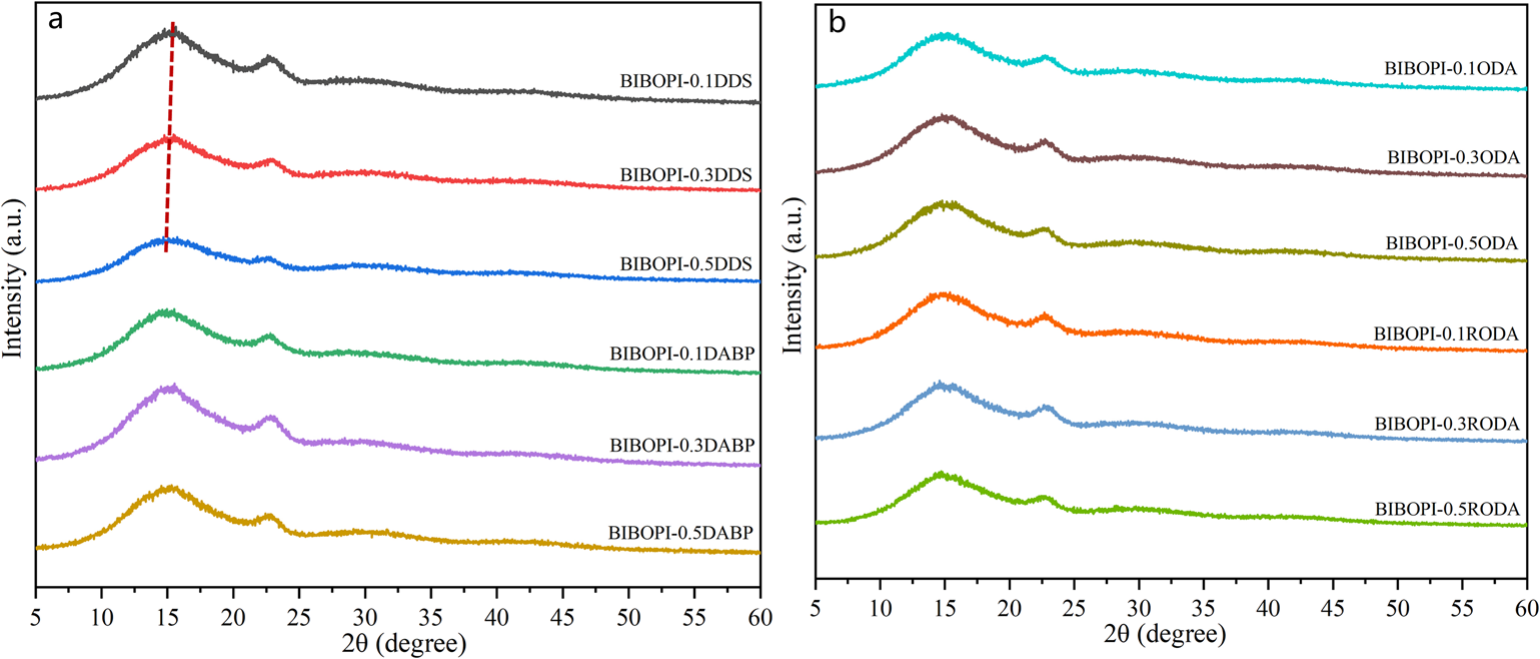
XRD curves of (a) BIBOPIs containing DDS and DABP diamine, (b) BIBOPIs containing ODA and RODA diamine.

X-ray diffraction analysis shown in Fig. 3a and b revealed amorphous characteristics in all BIBOPI films, evidenced by two broad diffraction peaks centered at approximately 15° and 22.5° corresponding to disordered main-chain packing.^34^ Bragg equation calculations showed distinct *d*-spacing variations between DDS- and RODA-incorporated samples. For DDS-modified films, the *d*-spacing progressively increased from 5.72 Å (BIBOPI-0.1DDS at 15.478°) to 5.86 Å (BIBOPI-0.5DDS at 15.104°), indicating that DDS incorporation induces chain relaxation and expanded interchain distances. Conversely, RODA-containing films exhibited an opposite trend with slightly reduced *d*-spacings from 6.03 Å (BIBOPI-0.1RODA at 14.683°) to 5.99 Å (BIBOPI-0.5RODA at 14.777°), suggesting that increased RODA concentration enhances molecular chain mobility and promotes more compact chain stacking. This contrasting behavior demonstrates the differential effects of DDS (chain separation) and RODA (chain reorganization) additives on polymer packing morphology.

For materials that provide protective functions, thermal stability is an essential property, as it determines the upper limit of the material's operational temperature. The long-term operating temperature of conventional soft-pack battery protective layers, such as polypropylene (PP), polyester (PET), and polyamide (PA), is typically below 150 °C, which fails to satisfy the protection requirements of high-temperature, high-energy-density, and high-performance batteries, including those used in aerospace, medical devices, industrial equipment, and high-load energy storage applications. *T*_g_ of BIBOPIs can be analyzed through DSC curve, as illustrated in Fig. S2a and b. All BIBOPI films exhibited exceptional thermal stability with *T*_g_ values exceeding 300 °C. Notably, samples containing DDS demonstrated significantly attenuated *T*_g_ reduction compared to other diamine-modified systems, attributed to dual mechanisms: (a) steric hindrance from DDS's sulfonyl groups restricts molecular chain mobility, and (b) robust hydrogen-bonding interactions form between electron-withdrawing sulfonyl moieties and electron-donating imidazole rings. Although DDS's folded molecular configuration increased polymer free volume, chain mobility remained constrained by rotational energy barriers inherent to its structural units. Conversely, the introduction of flexible ether bonds in ODA and RODA derivatives enhanced chain flexibility, resulting in a measurable reduction in *T*_g_, as manifested by BIBOPI-0.5ODA (335 °C) and BIBOPI-0.5RODA (326 °C), respectively, showing a decrease related to the concentration of ether bonds. This contrast highlights the tunable thermal properties achievable through strategic selection of diamine monomers, balancing chain rigidity (DDS) *versus* conformational freedom (ODA/RODA).

Thermogravimetric analysis of BIBOPIs under N_2_ and air atmospheres presented in Fig. S3a and b demonstrated exceptional thermal stability, with decomposition parameters systematically quantified in Table 1. In N_2_ atmosphere, the 5% (*T*_5%_) and 10% (*T*_10%_) weight loss temperatures spanned 532–575 °C and 559–599 °C, respectively, while maintaining 62–67% residual mass at 800 °C. In the air atmosphere, slightly reduced stability was observed with *T*_5%_ = 520–549 °C and *T*_10%_ = 544–569 °C. Notably, the reference polymer BIBO-BPDA (without flexible monomers) exhibited superior thermal stability (*T*_5%_ = 578 °C, *T*_10%_ = 600 °C in N_2_).^24^ Controlled incorporation of DABP and ODA at low concentrations preserved thermal integrity. BIBOPI-0.1DABP (*T*_5%_ = 575 °C) and BIBOPI-0.1ODA (*T*_5%_ = 571 °C) showed minimal degradation loss. DABP-modified systems displayed particularly robust performance, with BIBOPI-0.3DABP (*T*_5%_ = 571 °C) and BIBOPI-0.5DABP (*T*_5%_ = 569 °C) maintaining high thermal stability. In contrast, RODA incorporation introduced a flexibility-stability tradeoff. Even 0.1 mol% RODA reduced BIBO-BPDA's thermal stability by ∼17 °C, demonstrating the ether bond's dual role in enhancing chain mobility while compromising heat resistance through structural destabilization.

**Table 1:** Thermal property, coefficient of thermal expansion and intrinsic viscosity of BIBOPIs

Sample	Mole ratios	*T*_g_ (°C)		N_2_ (°C)		Air (°C)		*R*_800 °C_ (%)	CTE (ppm/°C)
	BIBO/X/BPDA	DSC	DMA	*T*_5%_	*T*_10%_	*T*_5%_	*T*_10%_		
BIBO-BPDA^a^	1/0/1	—	420	578	600	569	590	65	4.02
BIBOPI-0.1DDS	0.9/0.1/1	400	410	564	590	547	565	65	13.24
BIBOPI-0.3DDS	0.7/0.3/1	396	404	553	583	544	562	63	22.18
BIBOPI-0.5DDS	0.5/0.5/1	378	383	532	559	539	571	62	34.12
BIBOPI-0.1DABP	0.9/0.1/1	401	405	575	599	541	566	66	16.18
BIBOPI-0.3DABP	0.7/0.3/1	376	376	571	595	545	566	65	23.12
BIBOPI-0.5DABP	0.5/0.5/1	341	343	569	592	546	565	67	33.32
BIBOPI-0.1ODA	0.9/0.1/1	396	401	571	594	540	561	66	11.85
BIBOPI-0.3ODA	0.7/0.3/1	371	374	565	589	549	567	64	24.47
BIBOPI-0.5ODA	0.5/0.5/1	335	349	562	585	549	569	63	33.39
BIBOPI-0.1RODA	0.9/0.1/1	398	406	561	586	535	555	65	11.61
BIBOPI-0.3RODA	0.7/0.3/1	369	373	557	581	540	563	63	24.91
BIBOPI-0.5RODA	0.5/0.5/1	326	332	549	571	520	544	62	36.68

a Relevant data previously reported by the laboratory in the literature.^24^

The glass transition determination of benzoheterocycle PIs poses inherent challenges in DSC, a limitation shared among many high-temperature polymers due to subtle transition signals. DMA, with superior sensitivity to molecular mobility changes, provides more definitive thermal transition characterization for BIBOPI films. As illustrated in Fig. S4a and b, DMA-derived *T*_g_ values (identified as tan *δ* peaks) spanned 332–410 °C, systematically decreasing with third-monomer incorporation. The sulfone-containing diamine (DDS) exhibited the weakest *T*_g_-modifying effect, attributed to its dual constraints: strong polar interactions and steric hindrance from the sulfone group, which paradoxically restrict chain mobility despite increasing free volume. The storage modulus of the BIBOPIs exhibited a gradual decline during the glass transition, yet the magnitude of this change remained relatively consistent. The BIBOPI films exhibited restricted kinetic activity during the glass transition, which correlates with the inconspicuous *T*_g_ transition observed in the DSC curves. A low concentration of the third monomer (0.1–0.3 mol%) induced negligible modulus alterations near *T*_g_, while a high concentration of the third monomer (0.5 mol%) causes a significant modulus loss, which is related to the enhancement of the transition detectable by DSC. Notably, BIBOPI-0.5 systems displayed sharper *T*_g_ features in both DMA and DSC, underscoring the concentration-dependent activation of chain mobility mechanisms. This dual-method analysis elucidates the interplay between monomeric structure (polarity and flexibility), composition, and thermal transition detectability in high-performance PIs.

TMA analysis of the BIBOPI films presented in Fig. S5a and b revealed CTE values ranging from 11.61 to 36.68 ppm per °C within 50–250 °C (Table 1), demonstrating a systematic increase in CTE with higher incorporation ratios of the third-component diamine monomer. This trend is directly related to the reduction of dimensional stability, reflecting the key influence of molecular structure on thermal expansion behavior. Specifically, chain rigidity, conformational flexibility, and structural linearity (governed by the monomeric design) dictate both linear and volumetric expansion responses. While all four monomer types exhibited comparable CTE modulation below the *T*_g_, strategic adjustment of the third monomer's proportion enables precise tuning of the CTE to match substrate requirements. Notably, the compositional dependence of CTE highlights a design paradigm: increasing flexible/curved monomer content enhances chain mobility, thereby elevating thermal expansion, whereas rigid/linear components promote dimensional stability. This structure–property relationship establishes a rational framework for engineering BIBOPI films with substrate-compatible thermal expansion profiles through controlled ternary monomer integration.

Mechanical performances were investigated utilizing a universal testing machine. The stress–strain curves of the BIBOPI films are illustrated in Fig. S6a and b, with detailed data summarized in Table 2. The BIBOPI films exhibited tensile strengths ranging from 114 to 240 MPa, tensile moduli varying between 5.3 and 7.3 GPa, and elongation at break values spanning 4.5% to 33.8%. Notably, the incorporation of DDS led to a pronounced deterioration in mechanical properties: tensile strength decreased from 224 MPa (BIBOPI-0.1DDS) to 114 MPa (BIBOPI-0.5DDS), accompanied by a drop in elongation at break to 4.5%. This decline can be attributed to the relatively low reactivity of DDS, which limits the formation of high-molecular-weight polymer chains. The resulting reduction in molecular weight adversely affects mechanical strength by diminishing chain entanglement and stress transfer efficiency, consistent with established structure–property relationships in PIs.^35^ Conversely, increasing the RODA ratio led to a substantial increase in elongation at break from 6.8% (BIBOPI-0.1RODA) to 33.8% (BIBOPI-0.5RODA), indicating enhanced film toughness. This improvement can be explained by the –O– groups in the BIBOPI molecular chains, which increase molecular fluidity, expand interchain spacing, and weaken intermolecular interactions. These findings establish a monomer selection matrix for tailoring BIBOPI mechanical profiles to application-specific requirements.

**Table 2:** Mechanical properties of BIBOPIs

Sample	*σ* (MPa)	*E* (GPa)	*ε* (%)
BIBOPI-0.1DDS	224 ± 2	7.3 ± 0.2	8.2 ± 0.2
BIBOPI-0.3DDS	176 ± 3	6.5 ± 0.2	7.7 ± 0.5
BIBOPI-0.5DDS	114 ± 6	5.3 ± 0.3	4.5 ± 0.3
BIBOPI-0.1DABP	240 ± 3	8.2 ± 0.1	7.8 ± 0.4
BIBOPI-0.3DABP	205 ± 4	6.9 ± 0.2	12.2 ± 0.1
BIBOPI-0.5DABP	177 ± 2	6.7 ± 0.1	10.6 ± 0.3
BIBOPI-0.1ODA	203 ± 3	7.2 ± 0.1	5.9 ± 0.2
BIBOPI-0.3ODA	194 ± 3	6.9 ± 0.1	10.9 ± 0.2
BIBOPI-0.5ODA	173 ± 2	6.1 ± 0.2	13.6 ± 0.3
BIBOPI-0.1RODA	202 ± 3	7.1 ± 0.1	6.8 ± 0.2
BIBOPI-0.3RODA	198 ± 2	5.6 ± 0.2	13.0 ± 0.2
BIBOPI-0.5RODA	186 ± 2	6.9 ± 0.1	33.8 ± 0.4

**Fig. 4:**
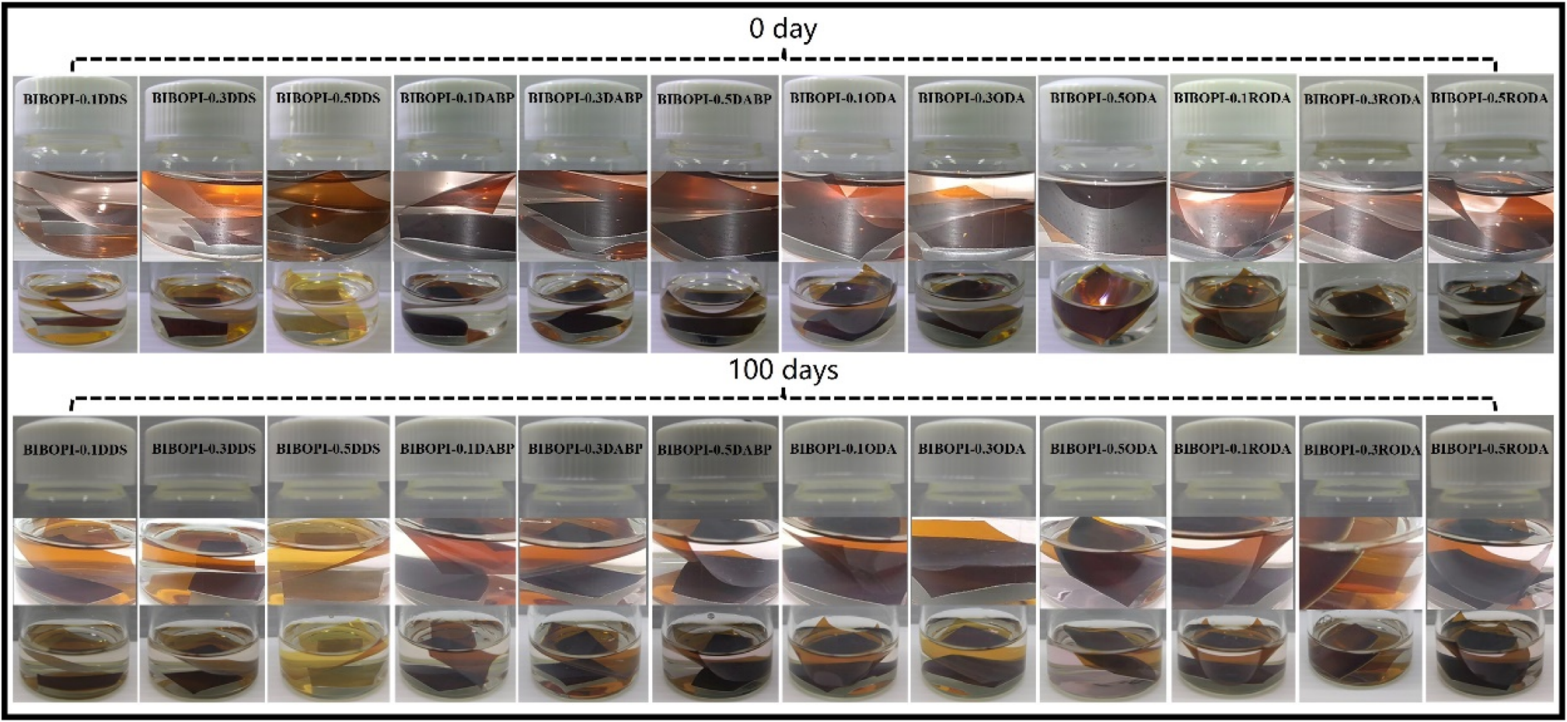
Electrolyte resistance of BIBOPI films and BIBOPI coated aluminum substrates.

The application environment of soft pack batteries frequently encounters significant challenges, such as acid fog intrusion and reaction liquid immersion. BIBOPI films must possess adequate solvent resistance to maintain the fundamental physical integrity. Systematic evaluation against common solvents, including Dimethylacetamide (DMAc), *N*-methylpyrrolidone (NMP), *m*-cresol, tetrahydrofuran (THF), dimethyl sulfoxide (DMSO), hydrogen fluoride (HF), and *N*, *N*-dimethylformamide (DMF), demonstrated complete stability of BIBOPI films, showing neither cracking nor swelling even under elevated temperatures. This chemical tolerance originates from robust hydrogen-bond networks formed by benzimidazole N–H groups within the polymer matrix,^36,37^ creating a molecular barrier against solvent penetration. In addition, long-term electrolyte resistance was assessed by fully immersing both free-standing BIBOPI films and BIBOPI-coated aluminum substrates (1 × 1 cm^2^) in a commercial electrolyte solution (Electrolyte for secondary lithium ion cell) for 100 days at 10–15 °C. As illustrated in Fig. 4, all samples maintain excellent morphological stability throughout the immersion period. Each image in Fig. 4 consists of two parts. The upper image provides a magnified view highlighting structural details, while the lower image shows the same sample at a macro scale. The magnified images clearly distinguish the two sample types: the upper segment corresponds to the free-standing BIBOPI film, and the lower segment shows the BIBOPI coating adhered to the aluminum substrate. Remarkably, the BIBOPI films showed no signs of dissolution, and the coated substrates exhibited intact adhesion with no warping, delamination, or peeling at the interface. The BIBOPI films did not dissolve in the electrolyte. The adhesion between the aluminum substrate and the BIBOPI coating was in excellent condition. No warping at the interface was observed, and there were no indications of delamination or peeling in the BIBOPI coating.

The *W*_A_ characteristics of BIBOPI films were systematically evaluated through 48 h immersion in deionized water, with results quantified in Table 3. A distinct inverse correlation emerged between third-component monomer content and *W*_A_ values, demonstrating that increased ternary monomer incorporation reduces hydrophilicity. This trend originates from the progressive dilution of BIBO's benzimidazole units, which decreases hydrogen-bonding probability between polymer chains and water molecules. The reduction in the content of low molar mass BIBO leaded to a decrease in the number of hydrogen bonds that can interact with water molecules within each unit mass segment of the polyimide film. Consequently, there were fewer adsorption sites for water molecules, resulting in a lower water absorption rate of the film. *W*_A_ values for 0.5 mol ratio composites followed a polarity-dependent hierarchy: BIBOPI-0.5DDS (1.3%) > BIBOPI-0.5DABP (0.9%) > BIBOPI-0.5ODA (0.8%) > BIBOPI-0.5RODA (0.5%). The observed progression aligns with the inherent polarity gradients of the third monomers-sulfone-containing DDS (highest polarity) exhibited maximal water affinity, while ether-rich RODA (lowest polarity) achieved optimal hydrophobicity. This structure–property relationship confirms that tailored monomer selection enables precise modulation of BIBOPI's moisture resistance through competitive hydrogen-bond disruption and polarity engineering.

**Table 3:** Water absorption, contact angle and adhesion grade of BIBOPIs

Sample	*W*_A_ a (%)	Contact angle (°)	Adhesion grade^b^	Adhesion^c^ time/h
BIBOPI-0.1DDS	2.5	51.06 ± 0.15	1	No shedding
BIBOPI-0.3DDS	2.5	48.68 ± 0.55	0	No shedding
BIBOPI-0.5DDS	1.3	38.25 ± 0.44	0	No shedding
BIBOPI-0.1DABP	2.2	48.94 ± 0.44	1	No shedding
BIBOPI-0.3DABP	1.3	47.55 ± 0.58	0	No shedding
BIBOPI-0.5DABP	0.9	37.13 ± 0.51	0	No shedding
BIBOPI-0.1ODA	2.2	53.63 ± 0.42	1	No shedding
BIBOPI-0.3ODA	1.6	48.15 ± 0.87	0	No shedding
BIBOPI-0.5ODA	0.8	42.74 ± 0.09	0	No shedding
BIBOPI-0.1RODA	1.4	55.44 ± 0.44	0	No shedding
BIBOPI-0.3RODA	1.3	49.97 ± 0.18	0	No shedding
BIBOPI-0.5RODA	0.5	44.15 ± 0.33	0	No shedding

a The sample was immersed in deionized water for 48 h at 25 °C.

b According to ISO 2409.

c In the boiling experiment, the BIBOPI coated aluminum substrate was immersed in distilled water at 80 °C under conditions of 1.0 atm and 100% relative humidity to evaluate the coating removal process. The time required for the BIBOPI coating to completely detach from the aluminum substrate (*T* ≥ 12 h) was meticulously recorded.

**Fig. 5:**
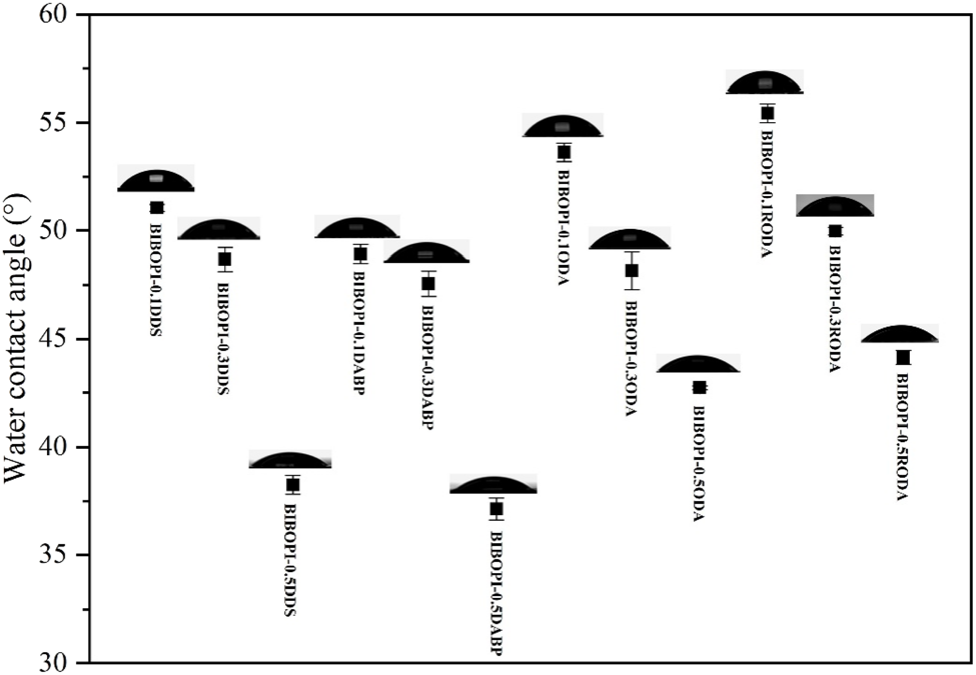
Contact angle of BIBOPIs (medium: water).

Surface wettability of BIBOPI films was quantitatively analyzed through water contact angle measurements shown in Fig. 5 and Table 3, revealing composition-dependent hydrophilicity trends. The introduction of the third component disrupted the structural regularity of the original BIBOPI film, enhancing the mobility of polyimide molecular chains. This leaded to a deterioration of its rigid structure, allowing polar or hydrophilic groups to migrate to the surface. Consequently, this resulted in an increase in surface energy and a reduction in water contact angle, thereby exhibiting improved wettability. In comparison to the polar third monomers DDS and DABP, ODA and RODA demonstrated relatively lower efficacy in reducing the contact angle. The contact angles for BIBOPI-0.5DDS and BIBOPI-0.5DABP were measured at 38.25° and 37.13°, respectively, whereas the contact angles for BIBOPI-0.5ODA and BIBOPI-0.5RODA were recorded at 42.74° and 44.15°, respectively. This discrepancy can be attributed to the substantial rise in surface energy of the films, which is primarily caused by their polar components.^38^

**Fig. 6:**
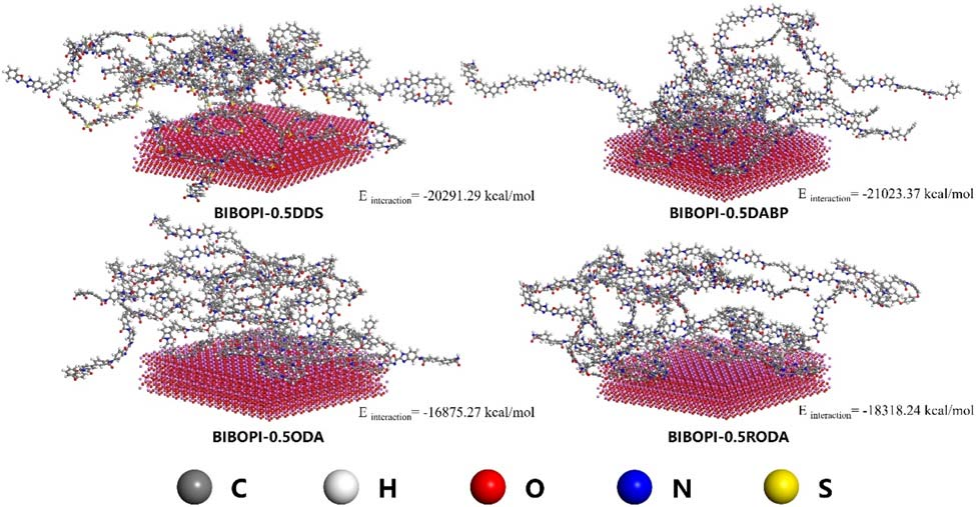
Simulation of interaction between BIBOPIs and substrates.

**Fig. 7:**
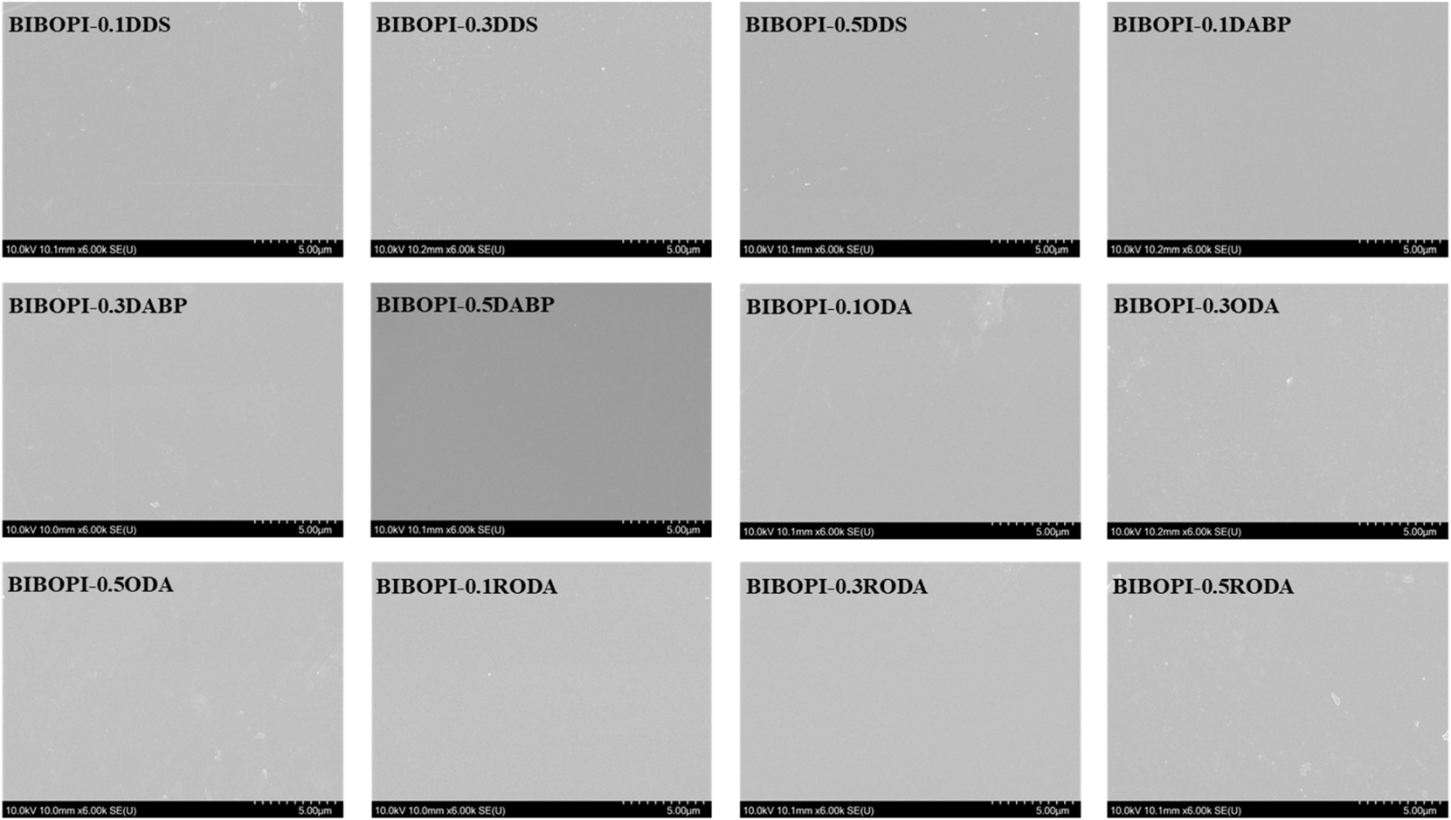
SEM of BIBOPI coated aluminum substrate.

Molecular dynamics (MD) simulations were employed to systematically evaluate interfacial adhesion mechanisms between BIBOPI coatings and aluminum substrates, utilizing representative 0.5 mol ratio third-component systems (Fig. 6). The optimized calculation showed that the interfacial energy of BIBOPI-0.5DDS (−20291.29 kcal mol^−1^), BIBOPI-0.5DABP (−21023.37 kcal mol^−1^), BIBOPI-0.5ODA (−16875.27 kcal mol^−1^) and BIBOPI-0.5RODA (−18318.24 kcal mol^−1^) were composition-dependent, where negative values denote thermodynamically favorable adhesion. These values indicated a tendency toward adhesion. Among them, the adhesion strength of BIBOPI-0.5RODA was significantly higher than that of BIBOPI-0.5ODA, as evidenced by the subsequent adhesion test experiments. The surface morphology of BIBOPI coatings on the aluminum substrate can be clearly observed through SEM analysis, as illustrated in Fig. 7. The incorporation of the flexible diamine component did not induce phase separation within the BIBOPI films. All BIBOPI films exhibited a dense and uniform surface morphology, free from observable pinholes, cracks, or other defects.

**Fig. 8:**
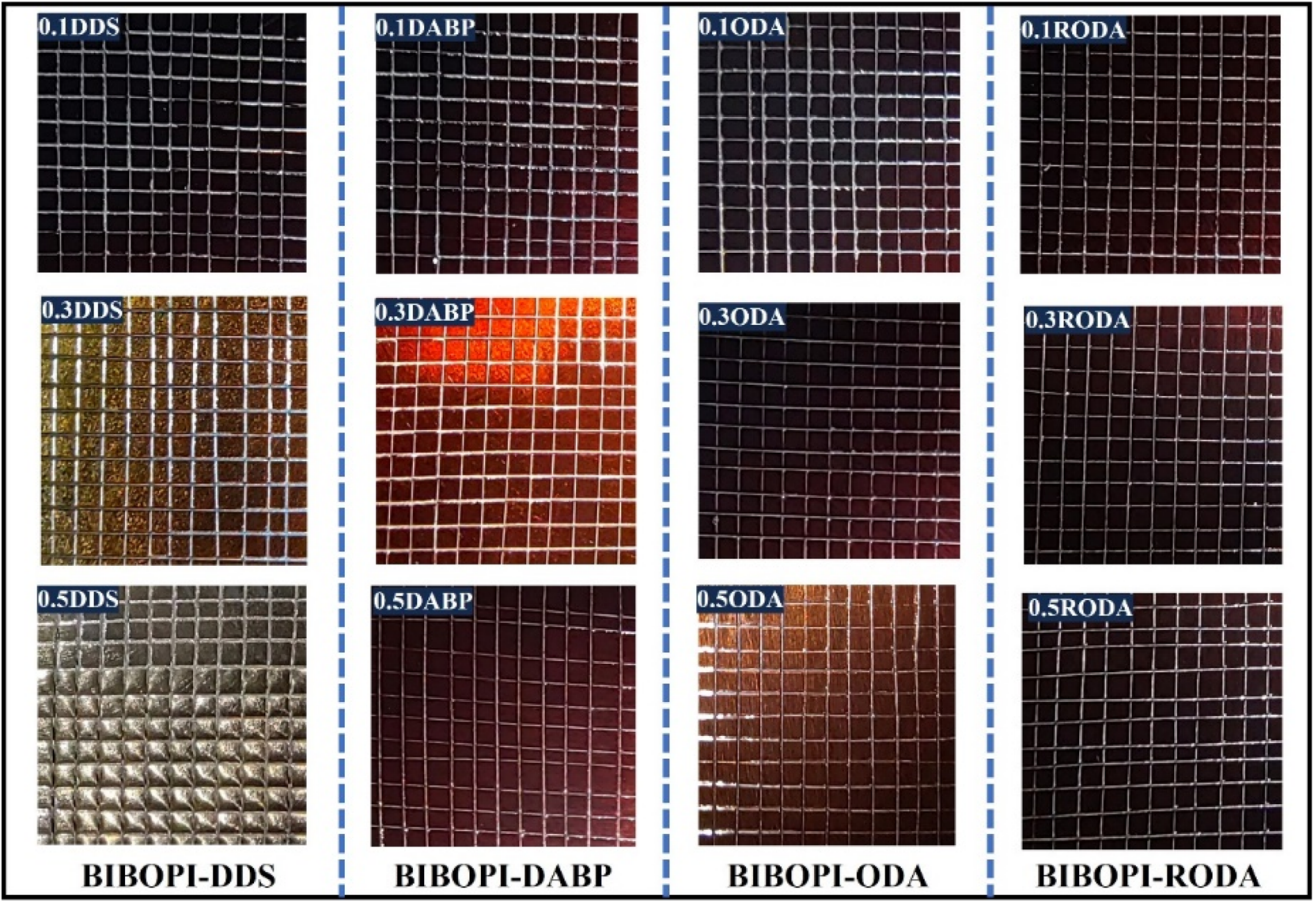
Test results of grid experiment (digital photos).

The adhesion of BIBOPI coatings to the aluminum substrates was quantitatively evaluated using the standardized grid test method in accordance with ISO 2409. Following a crosshatch incision and adhesive tape removal protocol, coating detachment patterns were analyzed. The results obtained from the grid method are illustrated in Fig. 8. Systems containing 0.1 mol ratio additives (BIBOPI-0.1DDS, BIBOPI-0.1DABP, and BIBOPI-0.1ODA) exhibited localized spalling at grid intersections, with ≤5% damage area corresponding to a grade 1 adhesion classification. It was evident that the incorporation of minor amounts of DDS, DABP, and ODA can significantly disrupt the inherent regularity of the coating. Notably, the residual BIBOPI coating demonstrated superior adhesion to the aluminum substrate (grade 0), characterized by smooth incision edges and negligible flaking, suggesting minimal disruption to coating integrity. These observations align with established adhesion theories encompassing chemical bonding, adsorption, electrostatic interactions, and mechanical interlocking.^39–42^ The excellent adhesion can be attributed to dual mechanisms. Firstly, the preferential bonding between BIBOPI coating and aluminum substrate facilitates the formation of metal–organic compounds *via* the C=O functional groups on the polymer.^20,21,23^ Secondly, the flexible molecular chain can be readily mechanically interlocked with the substrate, thereby enhancing the adhesion of the coating.^43^ Boiling experiment is an effective method for assessing the interfacial adhesion stability between BIBOPI coatings and aluminum substrates. The grid-patterned coating samples, prepared through the grid test, were fully immersed in ultrapure water maintained at 80 °C. The time-dependent interfacial integrity was systematically monitored by recording the precise duration until coating delamination occurred. As detailed in Table 3, the BIBOPI-coated specimens exhibited remarkable durability, with no observable coating detachment even after prolonged exposure exceeding 100 h under these accelerated hydrothermal conditions. This exceptional performance conclusively validates the superior interfacial adhesion stability of the BIBOPI coating system. While this study establishes a robust foundation through accelerated boiling-water testing, future work will focus on evaluating long-term adhesion durability under application-specific accelerated aging conditions, including cyclic humidity, UV exposure, and thermal cycling, to fully validate the performance of these materials in realistic battery packaging environments.

**Fig. 9:**
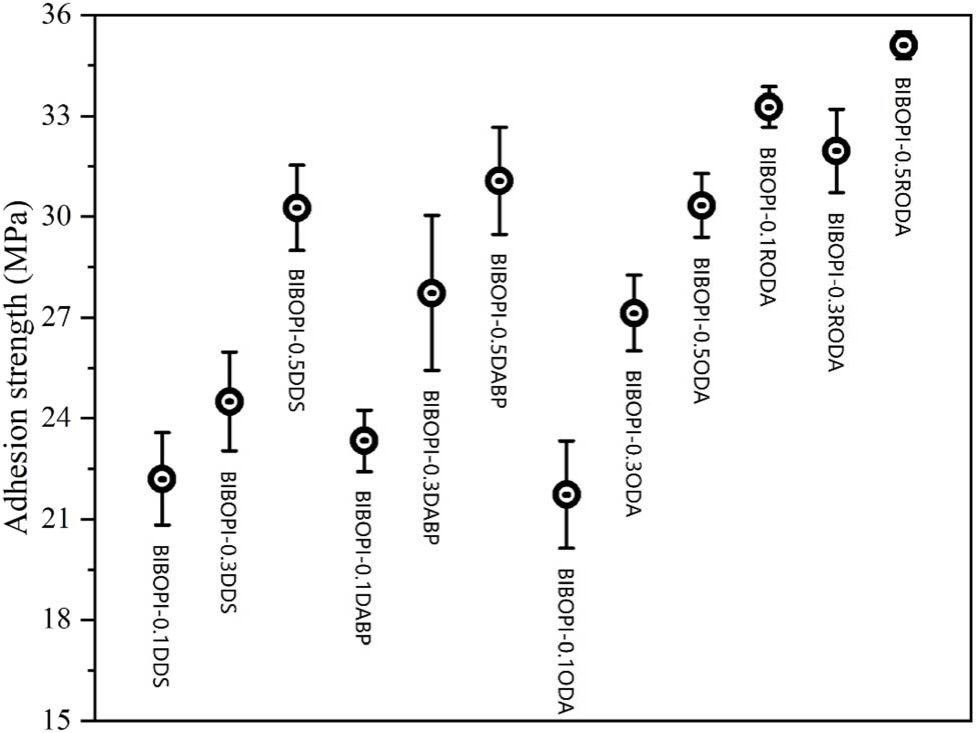
Influence of third monomer type and dosage on adhesion strength.

To further characterize interfacial adhesion performance, vertical pull-off testing was systematically implemented across modified BIBOPIs. As illustrated in Fig. 9, the strategic integration of flexible components substantially enhanced coating-substrate adhesion, achieving adhesion strengths surpassing 21.7 MPa. However, significant performance variations emerged among diamine-modified systems. While DDS-containing formulations demonstrated measurable improvements, for example, the adhesion strength of BIBOPI-0.3DDS at 24.5 MPa, their adhesion remained markedly inferior to DABP-incorporated counterparts (BIBOPI-0.3DABP at 27.7 MPa), attributable to DDS's inherent reactivity limitations. Comparative analysis revealed distinct structure–property relationships, with DABP's carbonyl groups enhancing molecular polarity and interfacial bonding capacity, while ODA-modified samples exhibited comparable flexibility but reduced adhesion strength at equivalent third-monomer loadings. Notably, RODA-incorporated systems demonstrated exceptional performance enhancement, with BIBOPI-0.1RODA achieving a remarkable 33.3 MPa adhesion strength, substantially exceeding values observed in DDS-, DABP-, and ODA-based components. These results highlight the critical role of monomer chemical architecture in optimizing both interfacial adhesion and mechanical interlocking capabilities.

**Fig. 10:**
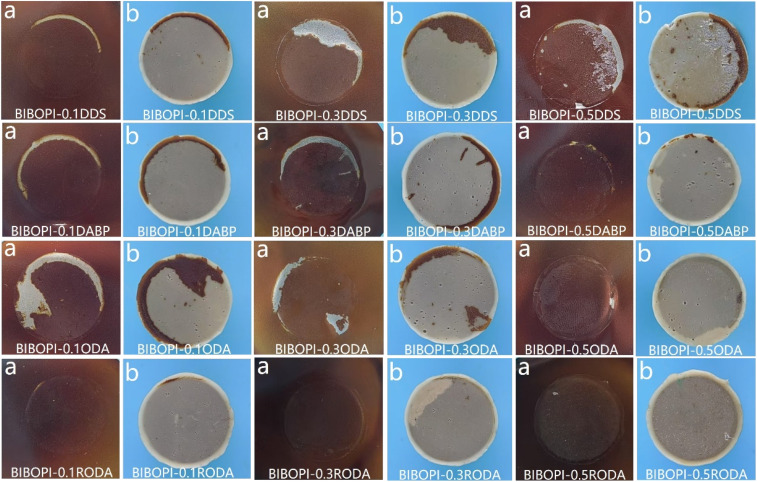
Test results of pull-off experiment (digital photos: (a) film interface; (b) spindle interface).

The morphological characteristics of the pull-out fracture surface are presented in Fig. 10. The BIBOPI-0.5DDS system exhibited mixed-mode failure characteristics, with approximately 40% residual adhesive layer retained on the coating surface, indicative of concurrent adhesive failure (coating-substrate interface) and cohesive failure (within adhesive matrix). This behavior stems from DDS's strong polarity and pronounced intermolecular interactions with adhesive constituents. In contrast, ODA-modified systems showed compromised structural integrity, where minimal ODA incorporation disrupted the densely crosslinked BIBO-BPDA network, creating localized weak interfaces that promoted stress concentration and reduced interfacial bond strength. Notably, RODA-incorporated systems (BIBOPI-0.1/0.3/0.5RODA) demonstrated exceptional interfacial stability, maintaining fully intact coating architectures without observable delamination. Their adhesion strengths exceeded the intrinsic adhesive-coating interfacial strength, a phenomenon attributable to RODA's molecular design advantages. This enhancement can be attributed to the highly flexible molecular structure of RODA, which significantly improved its infiltration capacity. The interaction between the flexible and rigid parts in the molecular chain forms a riveted structure with the irregular substrate surface, further enhancing the adhesion of the coating to the substrate.^43^

**Fig. 11:**
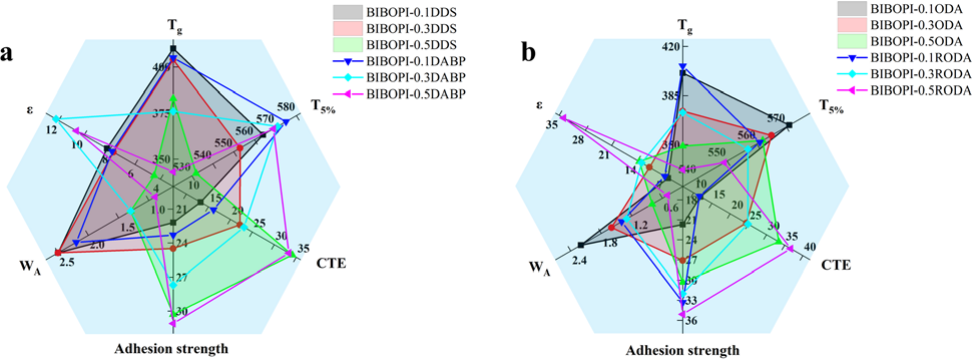
Comprehensive comparisons of BIBOPI samples properties: (a) BIBOPIs containing DDS and DABP diamine, (b) BIBOPIs containing ODA and RODA diamine.

The comprehensive properties of the prepared BIBOPI coatings were systematically evaluated, as illustrated in Fig. 11a and b. Among the polar component-containing BIBOPI films (Fig. 11a), those constructed with DDS exhibited superior heat resistance and dimensional stability. In contrast, BIBOPI-0.5DABP demonstrated lower water absorption, higher elongation at break, and enhanced adhesion strength. Notably, BIBOPI-0.3DABP exhibited the most balanced overall performance. As evident from Fig. 11b, BIBOPI-0.5RODA exhibited significantly greater elongation at break compared to other BIBOPI samples, with BIBOPI-0.3RODA showing relatively well-rounded comprehensive properties across multiple performance metrics.

## 4 Conclusions

A series of BIBOPI films were successfully synthesized through molecular chain engineering by incorporating third monomers with distinct structural features. Systematic characterization revealed key structure–property relationships. All modified systems exhibited reduced *T*_g_, with DDS-modified formulations showing the least pronounced *T*_g_ depression, indicative of its limited plasticizing effect. Mechanical flexibility was significantly enhanced in RODA-incorporated systems, where elongation at break increased proportionally with RODA content. All variants maintained exceptional solvent resistance across ambient and elevated temperatures, complemented by remarkably low *W*_A_ values of 1.3% (0.5DDS), 0.9% (0.5DABP), 0.8% (0.5ODA), and 0.5% (0.5RODA), respectively. Both free-standing films and aluminum-supported coatings demonstrated excellent morphological stability in electrolyte environments. Crucially, the adhesion performance improved with increasing flexible component content, achieving optimal interfacial bonding in highly modified systems without the need for complex surface treatments, such as magnetron sputtering, vapor deposition, hydrolysis, or plasma technology. These findings establish a materials design paradigm for next-generation polyimide-based aluminum-plastic flexible packaging, wherein molecular-level chain engineering circumvents the traditional reliance on heterogeneous adhesives while addressing environmental compatibility constraints.

## Author contributions

Feng Guo: writing – original draft, data curation, formal analysis. Wei Wang: data curation, formal analysis. Guangtao Qian: investigation, methodology. Dandan Li: methodology, writing – review & editing. Yongfeng Li: conceptualization, supervision, writing – review & editing.

## Conflicts of interest

The authors declare that they have no known competing financial interests or personal relationships that could have appeared to influence the work reported in this paper.

## Supplementary Material

RA-015-D5RA05693D-s001

## Data Availability

Data will be made available on request. Supplementary information is available. See DOI: https://doi.org/10.1039/d5ra05693d.
