# Supplementary material for: Tailoring substrate adhesion via flexible chain architecture design in benzoheterocycle polyimide protective coatings
Source: RSC Adv. 2025 Oct 20;15(47):39540–53. doi: 10.1039/d5ra05693d (PMC12536869; doi:10.1039/d5ra05693d)
Supplement: RA-015-D5RA05693D-s001 [file RA-015-D5RA05693D-s001.pdf]

Supplementary Information (SI) for RSC Advances.
This journal is © The Royal Society of Chemistry 2025

Supplementary Material

Tailoring substrate adhesion via flexible chain architecture design in benzoheterocycle polyimide protective coatings

Feng Guo[a], Wei Wang[b], Guangtao Qian[c], Dandan Li[a,d], Yongfeng Li[c]*

[a]State Key Laboratory of Advanced Fiber Materials, Center for Advanced Low-dimension Materials, College of Materials Science and Engineering, Donghua University, Shanghai, 201620, China

[b]School of Materials Science and Engineering, NingboTech University, Ningbo 315100, China

[c]Shanghai Collaborative Innovation Center of High Performance Fibers and Composites (Province-Ministry Joint), Center for Civil Aviation Composites, Donghua University, Shanghai, 201620, China

[d]School of Materials Science and Engineering, Shanghai University of Engineering Science, Shanghai 201620, PR China

*Corresponding authors E-mail: (Y. Li) yfli@dhu.edu.cn

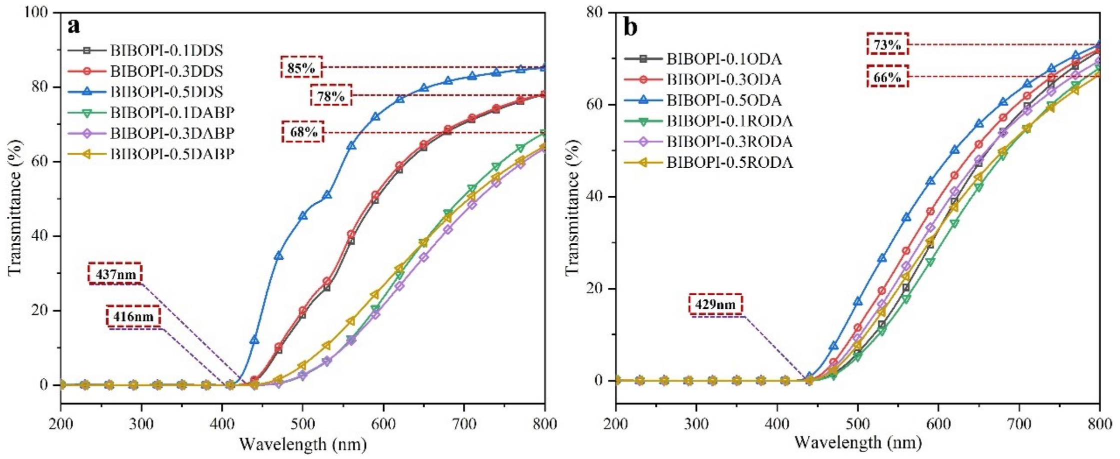

Fig. S1 UV-visible spectra of (a) BIBOPIs containing DDS and DABP diamine, (b) BIBOPIs containing ODA and RODA diamine.

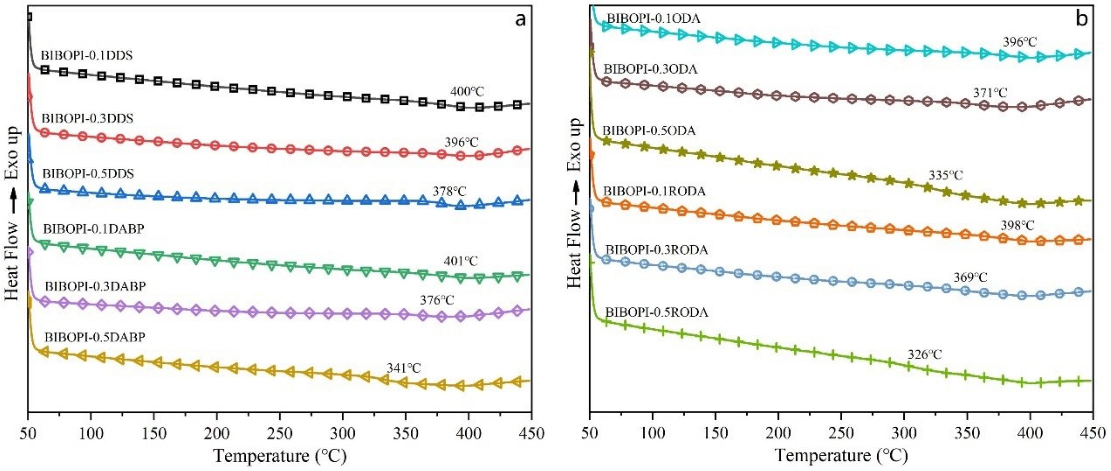

Fig. S2 DSC curves of (a) BIBOPIs containing DDS and DABP diamine, (b) BIBOPIs containing ODA and RODA diamine.

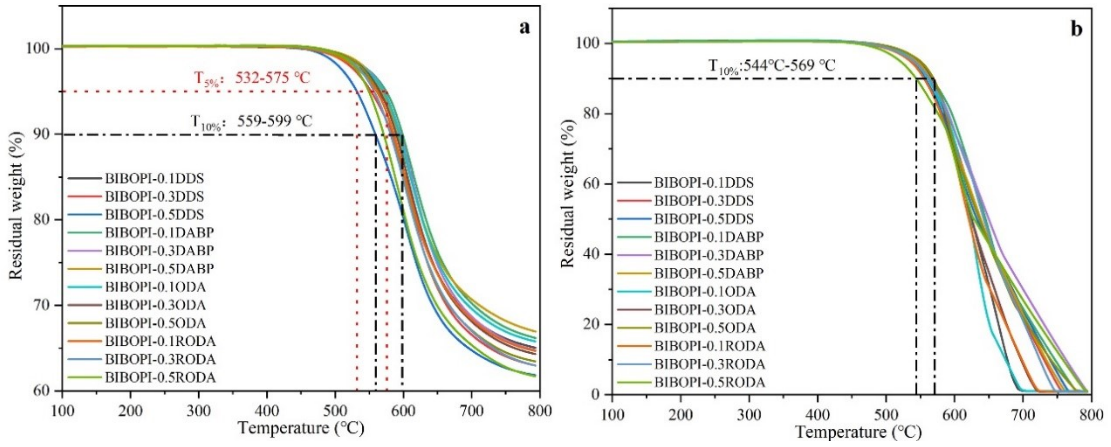

Fig. S3 TGA curves of BIBOPIs (a under N_2 atmosphere, b under air atmosphere)

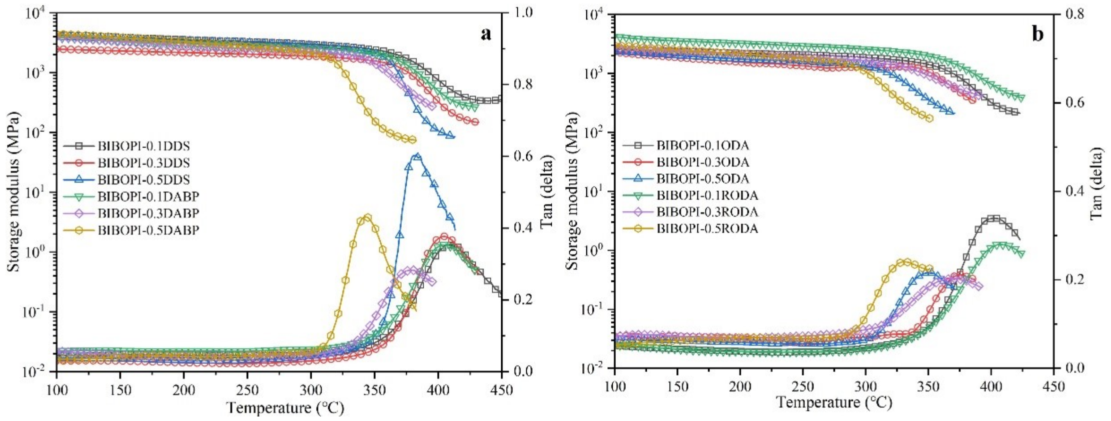

Fig. S4 DMA curves of (a) BIBOPIs containing DDS and DABP diamine, (b) BIBOPIs containing ODA and RODA diamine.

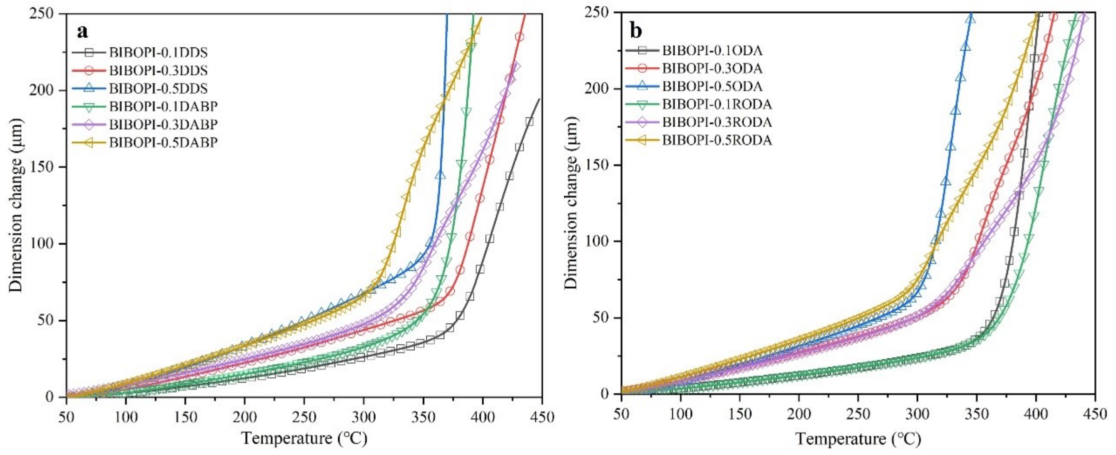

Fig. S5 TMA curves of (a) BIBOPIs containing DDS and DABP diamine, (b) BIBOPIs containing ODA and RODA diamine.

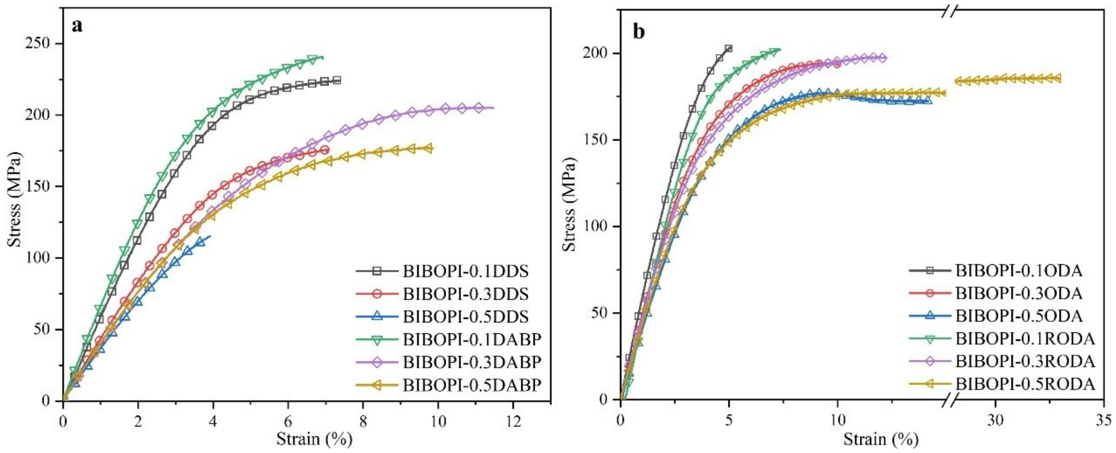

Fig. S6 Stress-strain curves of (a) BIBOPIs containing DDS and DABP diamine, (b) BIBOPIs containing ODA and RODA diamine.
